# Structure-function analyses of metal-binding sites of HypA reveal residues important for hydrogenase maturation in *Helicobacter pylori*

**DOI:** 10.1371/journal.pone.0183260

**Published:** 2017-08-15

**Authors:** Faith C. Blum, Heidi Q. Hu, Stephanie L. Servetas, Stéphane L. Benoit, Robert J. Maier, Michael J. Maroney, D. Scott Merrell

**Affiliations:** 1 Department of Microbiology and Immunology, Uniformed Services University of the Health Sciences, Bethesda, MD, United States of America; 2 Department of Chemistry and Program in Molecular and Cellular Biology, University of Massachusetts Amherst, Amherst, MA, United States of America; 3 Department of Microbiology, University of Georgia, Athens, GA, United States of America; Centre National de la Recherche Scientifique, Aix-Marseille Université, FRANCE

## Abstract

The nickel-containing enzymes of *Helicobacter pylori*, urease and hydrogenase, are essential for efficient colonization in the human stomach. The insertion of nickel into urease and hydrogenase is mediated by the accessory protein HypA. HypA contains an N-terminal nickel-binding site and a dynamic structural zinc-binding site. The coordination of nickel and zinc within HypA is known to be critical for urease maturation and activity. Herein, we test the hydrogenase activity of a panel of *H*. *pylori* mutant strains containing point mutations within the nickel- and zinc-binding sites. We found that the residues that are important for hydrogenase activity are those that were similarly vital for urease activity. Thus, the zinc and metal coordination sites of HypA play similar roles in urease and hydrogenase maturation. In other pathogenic bacteria, deletion of hydrogenase leads to a loss in acid resistance. Thus, the acid resistance of two strains of *H*. *pylori* containing a hydrogenase deletion was also tested. These mutant strains demonstrated wild-type levels of acid resistance, suggesting that in *H*. *pylori*, hydrogenase does not play a role in acid resistance.

## Introduction

*Helicobacter pylori* is a Gram-negative bacterium that colonizes the gastric mucosa of approximately 50% of humans [1]. Chronic infection with *H*. *pylori* is strongly associated with development of gastric carcinoma and B-cell mucosa-associated lymphoid tissue (MALT) lymphoma, leading to the classification of this bacterium as a Group 1 carcinogen [2]. Given the association with human disease, a greater understanding of the molecular mechanisms used by *H*. *pylori* to colonize the human stomach has the potential to reveal novel therapeutic targets, and thus, is of significant interest.

Two components that have been shown to be required for efficient colonization of *H*. *pylori* in animal models are the nickel-containing enzymes, urease and hydrogenase [3–8]. The importance of urease in *H*. *pylori* colonization and survival is intricately linked to the fact that *H*. *pylori* is not an acidophile and thus, must combat the low pH environment found in the stomach. This is partially accomplished by urease, which neutralizes the gastric microenvironment and buffers the bacterial periplasm and cytoplasm by catalyzing the conversion of urea into ammonia and carbon dioxide [9, 10]. The importance of urease in the *H*. *pylori* life cycle is evidenced by the fact that this enzyme represents approximately 10% of the total nascent protein in the cell [11].

At the molecular level, urease is composed of heterodimers of the structural subunits UreA and UreB, arranged in a dodecameric ((AB)_3_)_4_ structure [12], with two nickel ions bound by each dimer for a total of 24 nickel ions; therefore, urease represents a major nickel sink within *H*. *pylori* [13]. Indeed, nickel acquisition is crucial for *H*. *pylori*, which carries high-affinity transport systems to accommodate demand [14, 15]. Maturation of urease is accomplished with the aid of two clusters of accessory proteins. These include UreEFGH, which are required for the biosynthesis of the enzymatic metallocenter [16], and HypAB [17], which are also involved in hydrogenase maturation. The function of HypAB in urease maturation has been predicted from several biochemical studies that have demonstrated interactions between the Hyp and Ure pathways; HypA interacts with UreE [13] and competes with UreG for binding to UreE [18], and HypB interacts with UreG, UreA, and UreB [19]. Interestingly, the dependence of urease maturation on HypAB can be rescued with the addition of excess nickel [17]. Thus, the available data suggest that the contribution of HypAB to urease maturation likely lies in delivery of nickel to the UreEFGH pathway, which would help to compensate for the low nickel-binding ability of UreE [20].

**Fig 1:**
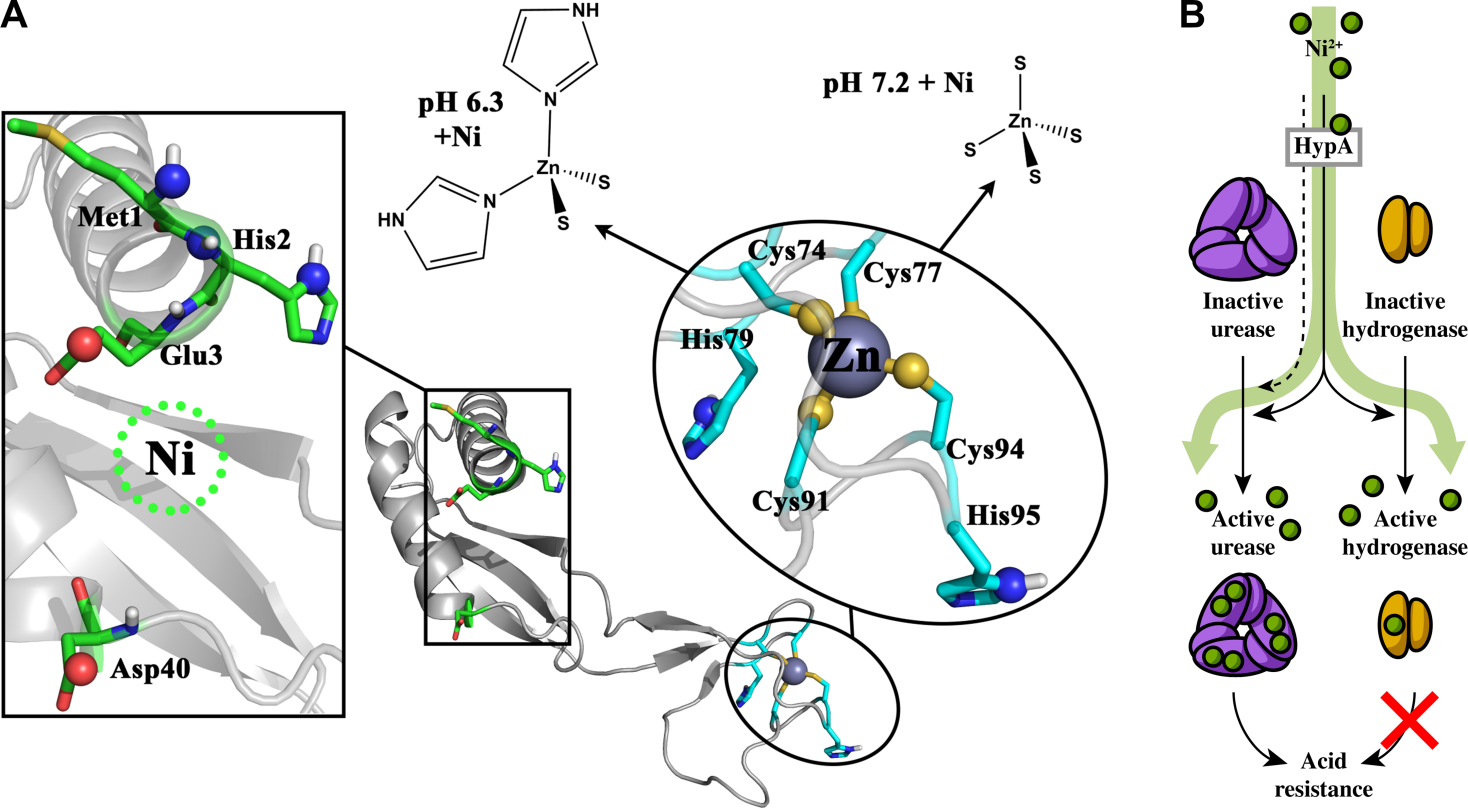
The structure of HypA and its role in urease and hydrogenase maturation. (A) Representation of the NMR structure of *H*. *pylori* HypA (PDB: 2KDX) [25] with the main chain colored in light grey and the metal binding sites in color to highlight the location of residues involved in metal coordination. Residues comprising the nickel-binding site (M1, H2, E3, and D40) are shown in green. Residues of the zinc-binding site (C74, C77, H79, C91, C94, and H95) are shown in cyan. The metal-binding oxygen (red), nitrogen (blue), and sulfur (yellow) atoms are shown as small spheres. The nickel atom representation in this figure (dotted green circle) was not resolved in the 2KDX structure, and the resolved zinc atom is shown as a dark grey sphere. The zinc-binding site adopts two pH-dependent conformations, as illustrated: Zn(Cys)_2_(His)_2_ at acidic pH, and Zn(Cys)_4_ at neutral pH. (B) HypA contributes to the maturation of hydrogenase and urease through delivery of nickel (green circles). Urease requires nickel for activity, of which one of the downstream effects is acid resistance. In the absence of HypA, maturation of urease can still be accomplished through the addition of excess nickel (dashed line). Hydrogenase requires nickel for activity, but herein is shown not to contribute to *in vitro* acid resistance (red X). In the absence of HypA, maturation of hydrogenase cannot be accomplished through the addition of excess nickel.

The second nickel-containing enzyme in *H*. *pylori* is the [NiFe] H_2_-uptake type hydrogenase. This hydrogenase is a heterotrimeric complex composed of HydA, the small subunit that contains multiple [Fe-S] clusters; HydB, the large subunit that contains the [NiFe] site; and HydC, a membrane-anchored cytochrome *b* [21]. Deletion of *hyd* results in attenuation in animal models [7, 8], which was attributed to the inability of the mutant strains to use H_2_ as an energy source within the animal [7]. As with urease, accessory proteins are involved in hydrogenase maturation; these include HydDE and HypABCDEF [17, 22]. The functions of HydD and HydE are not fully understood, but a considerable amount of effort has gone into structure-function analysis of HypA. Those studies have shown that HypA contains two metal binding sites: a low affinity nickel-binding site at the N terminus, and a high affinity structural zinc-binding site located near the C terminus (Fig 1A, green residues). HypA binds one nickel ion with micromolar *K*_d_ [23, 24]. The 6-coordinate nickel-binding site requires His2 [23], which participates through both the side chain imidazole and the backbone amide [25], and the N-terminal amine on Met1 [26]. The remaining nickel ligands are likely the side chains of Glu3 and Asp40, and a backbone amide [26]. At the C terminus, four Cys residues in two CxxC motifs form a tetrahedral zinc-binding site [25, 27] (Fig 1A, cyan residues). The zinc site may be involved in pH-sensing, as a decrease in pH from 7.2 to 6.3 changes the Zn(Cys)_4_ site to a Zn(Cys)_2_(His)_2_ site, using two His residues located immediately C-terminal to each CxxC motif [24]. The decrease in pH and switch to Zn(Cys)_2_(His)_2_ also corresponds to a decrease in the affinity of HypA for nickel [24]. Of note, in contrast to the complete rescue observed for the urease pathway, upon deletion of HypAB, hydrogenase maturation is only partially rescued with the addition of nickel [17]. This functional difference led us to question whether the HypA residues critical for urease maturation and hydrogenase maturation differ. Herein, we investigate this possibility as well as the role of hydrogenase in low pH resistance of *H*. *pylori*.

## Materials and methods

### Bacterial growth

Strains are listed and described in Table 1. *H*. *pylori* strains were maintained at -80°C in brain heart infusion (BHI) broth (BD) supplemented with 20% (v/v) glycerol (CalBioChem) and 10% (v/v) fetal bovine serum (FBS, Gibco). *H*. *pylori* G27 and G27-derived strains were grown on 4.4% (w/v) Columbia agar (Acumedia) supplemented with 5% (v/v) horse blood (HemoStat), 0.2% (w/v) β-cyclodextrin (Sigma), 10 μg/mL vancomycin (Amresco), 2.5 U/mL polymyxin B sulfate (Sigma), and 8 μg/mL amphotericin B (Amresco). Liquid growth of G27 strains was accomplished in Brucella broth (Acumedia) supplemented with 10% (v/v) FBS and 10 μg/mL vancomycin. G27 cultures were grown at 37°C under microaerophilic conditions (10% CO_2_, 5% O_2_, and N_2_ as balance) achieved using an Anoxomat (Advanced Instruments Inc). *H*. *pylori* 26695 and 26695-derived strains were grown on Brucella agar plates (Becton Dickinson) supplemented with 10% sheep blood (Hemostat) in CO_2_ incubators (Thermo Scientific) under microaerophilic conditions (5% CO_2_, 4% O_2_, and N_2_ as balance). Liquid growth of 26695 strains was performed in BHI broth supplemented with 0.4% β-cyclodextrin (Sigma) under microaerophilic conditions (5% CO_2_, 5% O_2_, and N_2_ as balance). For both strains, selection was performed with 25 μg/mL kanamycin (Gibco) or 30 μg/mL chloramphenicol (Sigma), as appropriate. Liquid *H*. *pylori* cultures were grown with shaking at 110 RPM. *Escherichia coli* Top10 cells were grown at 37°C on 4% LB agar plates (MoBio) or in liquid in LB broth (Invitrogen) with shaking at 225 RPM. Selection was performed with 100 μg/mL ampicillin (Affymetrix).

**Table 1:** Strains, plasmids, and primers used in this study.

**Strains**	**Description**	**Reference**
DSM1	G27 WT	[28]
DSM43	G27 Δ*ureB* (Δ*ureB*::*kan*), Kan^R^	[29]
DSM1283	G27 *hypA*::*kan-sacB*, Kan^R^	[30]
DSM1295	G27 *hypA* restorant	[30]
DSM1296	G27 *hypA* C74A	[30]
DSM1297	G27 *hypA* C94D	[30]
DSM1298	G27 *hypA* C91A	[30]
DSM1299	G27 *hypA* C91D	[30]
DSM1300	G27 *hypA* H95A	[30]
DSM1301	G27 *hypA* C74D	[30]
DSM1363	G27 *hypA* C77A	[30]
DSM1364	G27 *hypA* C77D	[30]
DSM1365	G27 *hypA* H79A	[30]
DSM1366	G27 *hypA* C94A	[30]
DSM1475	G27 *hypA* L2*	[26]
DSM1569	*E*. *coli* TOP10 containing pDSM1569, Kan^R^	This study
DSM1570	G27 Δ*hydB* (Δ*hydB*::*kan*), Kan^R^	This study
26695	WT	[31]
SLB1333	26695 Δ*ureAB* (Δ*ureAB*::*cat*), Cm^R^	This study
SLB1166	26695 Δ*hydABCDE* (Δ*hydABCDE*::*cat*), Cm^R^	[32]
**Plasmids**	**Description**	**Reference**
pDSM199	pTM117 vector with Kan^R^ gene	[33]
pDSM1569	pGEM T-Easy vector with KanR gene fused with sequence upstream and downstream of *hydB*	This study
**Primers**	**Sequence (5′-3′)**	**Reference**
hydB_us_F	GCAATGTGCTTTATTACTTGATG	This study
hydB_us_R	GTTAGTCACCCGGGTACCGAGCTCGACATGTTAATCCCTTACTCTTTG	This study
hydB_ds_F	CTAGAGTCGACCTGCAGGCATGCAAGGGACACGCATGGATAAAATG	This study
hydB_ds_R	GGTTATGGTTATACCAAAGAATGA	This study
NP_kan_F	GAGCTCGGTACCCGGGTGACTAACTAGGAGGAATAAATG	[34]
NP_kan_R	CTTGCATGCCTGCAGGTCGACTCTAGAGGATCCCCGGGTCATTATTCCCTCCAGGTACTA	[34]
hydB_conf_F	CCAGTTAAGGAGTGGCG	This study
hydB_conf_R	GGATATTTCCAATGCCTAAAATTAG	This study
ureABR2	TCCCTAAAGGGATTTTCAAGATGT	[35]
ureCAMF2	CCCAGTTTGTCGCACTGATAACCATGTGTTCGTGGATGGCAA	[35]
C1	GATATAGATTGAAAAGTGGAT	[35]
C2	TTATCAGTGCGACAAACTGGG	[35]
ureCAMR1	ATCCACTTTTCAATCTATATCATTCTCCTATTCTTAAAGTGTTTT	[35]
ureABF1	CATGGGGGCGTGGTGGATTA	[35]

### Δ*hydB* and Δ*ureAB* mutant construction

A Δ*hydB* deletion insertion strain containing a *kan* cassette replacing the coding sequence for *hydB* (HPG27_ RS03075) was constructed as follows. *H*. *pylori* G27 (Table 1) genomic DNA was used as a template for polymerase chain reaction (PCR) to amplify fragments of DNA flanking *hydB*. The primer pair hydB_us_F and hydB_us_R was used to amplify a 490-bp fragment upstream of *hydB* (containing coding sequence for *hydA*). The upstream primer contained an additional 24-bp complementary sequence to the 5′ sequence of the *kan* cassette. The primer pair hydB_ds_F and hydB_ds_R was used to amplify a 683-bp fragment downstream of *hydB* (containing coding sequence for *hydC*); the forward primer contained an additional 26-bp sequence that was complementary to the end of the *kan* cassette. Primer pairs NP_kan_F and NP_kan_R were used to amplify *aphA3*, which encodes a Kan^R^ gene, from pDSM199. The three fragments were assembled using splicing-by-overlap-extension (SOE) PCR. The spliced PCR product was ligated into the pGEM-T Easy vector (Promega) and transformed into *E*. *coli* TOP10 cells. Transformants were selected with ampicillin and screened for growth of white colonies on X-gal and IPTG. The desired SOE product was confirmed by Sanger sequencing. The plasmid containing the sequenced product was named pDSM1569, and the *E*. *coli* strain containing pDSM1569 was named DSM1569. The 2061-bp SOE product was amplified from *E*. *coli* using primers that annealed to the backbone of the plasmid, and 500 ng of the resulting linear DNA was transformed into *H*. *pylori* G27. Transformants were selected for on HBA plates containing kanamycin. Incorporation of the *kan* cassette to replace *hydB* was confirmed by Sanger sequencing using hydB_conf_F and hydB_conf_R primers. The resultant mutant strain was named DSM1570 (Table 1). A Δ*ureAB* deletion insertion strain containing a *cat* cassette replacing the coding sequence for *ureAB* (HP0073 and HP0072) was constructed in *H*. *pylori* 26695 using a SOE PCR method as described previously [35].

### Hydrogenase activity

Hydrogen oxidation activity was measured at room temperature (~23°C) using whole cell lysates of various *H*. *pylori* strains. Cells were grown as previously described [30] and approximately 10^8^ cells were pelleted and frozen at -80°C prior to lysis. Upon thawing, cells were resuspended in 750 μL of ice-cold *H*. *pylori* lysis buffer (50 mM HEPES at pH 7.0 with 1 mM phenylmethansulfonyl fluoride (PMSF)) and then kept on ice during lysis by pulsed sonication (500 Hz) at 40% amplitude for 14 seconds. H_2_ consumption was measured as previously described [36] with slight modifications. Briefly, an aliquot of whole cell lysate was used to test H_2_ oxidation activity by monitoring the reduction of methyl viologen (MV) inside an anaerobic chamber (COY Labs, Grass Lake, MI) with a consistent atmosphere of primarily N_2_ with 3–4% H_2_ and 0–20 ppm O_2_. Oxidized-MV (colorless) is the electron acceptor in the H_2_ oxidation reaction catalyzed by hydrogenase in the lysate resulting in reduced-MV (blue). The appearance of reduced-MV was monitored at A_578 nm_ (ε = 9.78 mM^-1^ • cm^-1^) [37] where two reduced-MV were expected for every H_2_ oxidized [36]. The reaction was initiated by adding 30 μL of whole cell lysate to 1 mL of deoxygenated H_2_ase Reaction Buffer (50 mM Tris-HCl, 2 mM methyl viologen, pH 8.0) in a glass vial. After an initial lag time, the reaction mixture was transferred to a quartz cuvette and the appearance of methyl viologen reduction was monitored at A_578 nm_ for 45–150 minutes, of which the slope of the linear increase in absorbance over time is taken as the rate. The lag is due to anaerobic activation that occurs upon exhaustion of trace oxygen levels [38]. Reaction initiated by lysis buffer alone was used to correct for background activity/instrument drift, which is in the same order of magnitude (H_2_-OX ~10^−8^ μmol/min) as the Δ*hydB* strain (where no activity was expected) and less than 1% of any other strains tested. The specific H_2_-oxidation activity was obtained by normalizing against the total protein in the whole cell lysate. Three biological replicates were tested for each strain (unless otherwise noted) and the specific H_2_-oxidation activity of each strain was normalized against the activity of the wild-type strain to obtain percent hydrogenase activity relative to wild-type.

### Urease activity

Urease activity assays of whole cell extracts of *H*. *pylori* wild-type, Δ*ureB*, and Δ*hydB* strains were performed as previously described [30]. Briefly, for each strain, overnight liquid cultures of *H*. *pylori* were used to inoculate 5 mL liquid cultures to an optical density at 600 nm (OD_600_) of 0.05, and then allowed to grow for 22 hr. At that point, the OD was measured and 1 OD unit of *H*. *pylori* was pelleted by centrifugation. The supernatants were removed and the bacterial pellets were stored at -20°C until ready for urease assays. The frozen cells were thawed and resuspended in 750 μL of ice-cold lysis buffer (HEPES buffer at pH 7.0, 1 mM PMSF, and 1x protease inhibitor cocktail). Resuspended cells were kept on ice during lysis, which was performed by sonication at 70% power for 6 pulses (2 sec each). The lysate was centrifuged at 15,000 x *g* for 10 min to remove insoluble material from the soluble whole cell extracts. The protein concentration of soluble whole cell extracts was determined by Bradford assays using the Pierce Coomassie Protein Assay Kit (Thermo Fisher Scientific). Urease activity was determined using a modified phenol-hypochlorite method, which measures the amount of ammonia released from the soluble whole cell extract in the presence of urea [39, 40]. Five μL of whole cell extract was added to 245 μL of urease reaction buffer (50 mM HEPES, 25 mM urea, at pH 7.0), and incubated at 37°C for 20 min to allow for ammonia production. The reaction was quenched by the sequential addition of 375 μL of phenol-hypochlorite buffer A (100 mM phenol, 167.8 mM sodium nitroprusside) and of 375 mL of phenol-hypochlorite buffer B (125 mM NaOH, 0.044% NaClO). The assay mixture was incubated at 37°C for 30 minutes to allow for color development, due to the conversion of phenol to indophenol, and measured at A_625 nm_. Assays were performed alongside a standard curve created using known amounts of ammonium chloride in place of the whole cell extract. The specific urease activity was determined by calculating the nmol of ammonia produced per μg of total protein in each whole cell extract. Relative urease activity of each strain was determined by normalizing against the specific activity of the wild-type strain to obtain percent urease activity relative to wild-type. Three biological replicates were tested for each strain.

### Acid survival assay

Liquid cultures of *H*. *pylori* G27 were inoculated to an OD_600_ of 0.05 from an overnight starter liquid culture and were then grown for 18–19 h to an OD_600_ of approximately 0.9–1.0. 1-mL aliquots were pelleted at 2500–3000 x *g* and were resuspended in 1 mL phosphate buffered saline (PBS) at pH 6.0 or pH 2.3, and with or without supplemented 5 mM urea. To prepare the resuspension solutions, urea was added from a freshly made 100 mM (in PBS) stock, as appropriate, and the pH of the solutions was adjusted using 6 M HCl. Immediately after resuspension, a 100-μL aliquot of *H*. *pylori* was removed, serially diluted in Brucella broth to 10^−7^, and 10 μL aliquots of each dilution were plated to determine the colony-forming units (CFU) at T_0_ for each condition. The remaining *H*. *pylori* were incubated at 37°C for 1 h with shaking. At 1 h, a second 100-μL aliquot was removed, serially diluted, and plated to determine the CFU at T_60_ for each condition. CFU counts were quantified after 4–5 d of growth. A similar protocol was used to monitor acid survival of the 26695 strains with the following minor exceptions. Liquid cultures of 26695 wild-type and mutant strains were grown for 26–30 h to an OD_600_ of approximately 0.34–0.42. Cells were serially diluted up to 10^−5^ in PBS at pH 7.3 and 10 μL of the 10^−1^–10^−5^ dilutions was plated in triplicate. Percent survival was calculated for each strain and condition using the equation T_60_ / T_0_ x 100%. Three biological replicates were performed for each isolate and for each strain background.

### Data processing

Hydrogenase activity, urease activity, and acid survival data presented in Table 2 were processed as follows. The raw data was retrieved from the current study, Johnson *et al*. [30], and Hu *et al*. [26]. The urease activity presented in Johnson *et al*. had previously been normalized such that *hypA*-R = 100%; to match the data presented in the current study and in Hu *et al*., the raw data was renormalized such that WT = 100%. Between the current study and the previous publications, the urease activity and acid survival of two strains (WT and Δ*ureB*) had been reported three times, and for two additional strains (*hypA*::*kan-sacB* and *hypA*-R) had been reported two times. Similarly, the hydrogenase activity of these four strains was measured three or two times in the current study. To present data from multiple experiments with minimal bias, these values were averaged and the data are shown in Table 2.

**Table 2:** Hydrogenase activity, urease activity, survival at pH 2.3 with urea, and dissociation constants.^a^

Strain	Hydrogenase activity (mean ± SD, %)	Urease activity (mean ± SD, %)	Survival at pH 2.3 + 5 mM urea (mean ± SD, %)	*K*_D_ of HypA-Ni (mean ± SD, μM)
WT	100 ± 8	100 ± 30	60 ± 10	1.0 ± 0.2^c^
Δ*ureB*	150 ± 8	0.7 ± 0.1	<0.0001	ND^d^
*hypA*::*kan-sacB*	12 ± 1	1.2 ± 0.4	4 ± 2	ND
*hypA*-R	90 ± 20	50 ± 20	110 ± 20	ND
C74A	12 ± 5	2.1 ± 0.3^b^	70 ± 20^b^	54 ± 4^e^
C74D	15 ± 2	2.0 ± 0.4^b^	14 ± 10^b^	ND
C77A	36 ± 4	2.9 ± 0.9^b^	90 ± 50^b^	48 ± 2^e^
C77D	43 ± 4	5 ± 3^b^	90 ± 30^b^	ND
H79A	90 ± 20	30 ± 10^b^	90 ± 20^b^	3.2 ± 3^e^
C91A	12 ± 4	1.5 ± 0.3^b^	1.9 ± 0.9^b^	22 ± 3^e^
C91D	40 ± 10	5 ± 3^b^	90 ± 30^b^	ND
C94A	9 ± 4	2.3 ± 0.2^b^	1 ± 2^b^	16 ± 2^e^
C94D	60 ± 10	5 ± 2^b^	110 ± 50^b^	22 ± 2^e^
H95A	100 ± 30	100 ± 100^b^	80 ± 20^b^	2.1 ± 2^e^
L2*	14 ± 1	0.3 ± 0.5^c^	1.1 ± 0.5^c^	59 ± 12^c^
Δ*hydB*	0.06 ± 0.06	112 ± 8	65 ± 9	ND

^a^ Values shaded dark grey are indicative of a severe defect, defined as ≤15% hydrogenase activity, ≤2.5% urease activity, or ≤10% acid survival. Values shaded light grey are indicative of a moderate defect, defined as >15% and ≤60% hydrogenase activity; >2.5% and ≤10% urease activity; or >10% and ≤50% acid survival.

^b^ Johnson *et al*. [30].

^c^ Hu *et al*. [26].

^d^ ND (not determined).

^e^ Herbst *et al*. [24].

## Results

### *H*. *pylori* hydrogenase does not contribute to acid resistance

**Fig 2:**
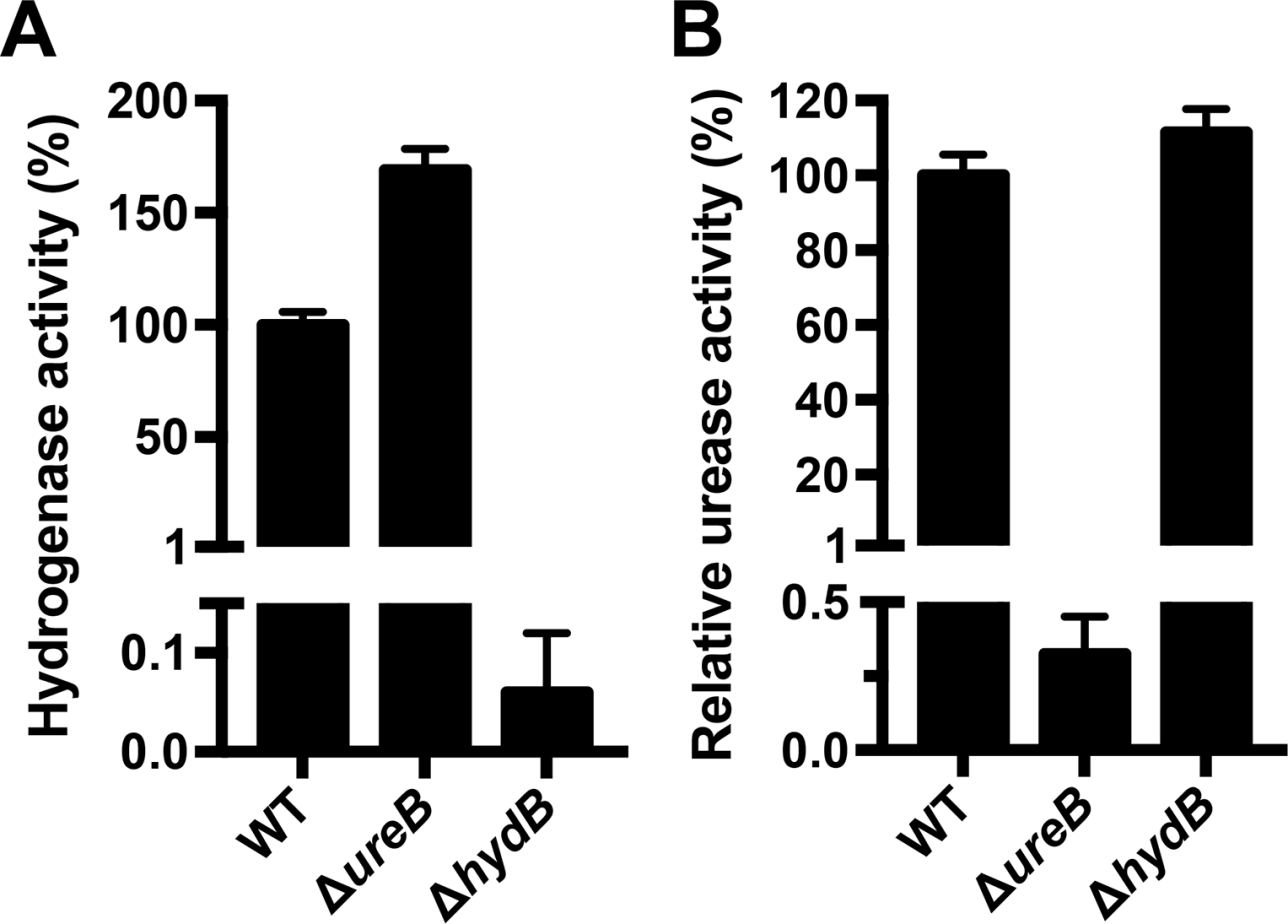
*H*. *pylori* Δ*hydB* has no detectable hydrogenase activity and wild-type urease activity. (A) Cell lysates of the wild-type (WT) strain, urease mutant strain (Δ*ureB*), and hydrogenase mutant strain (Δ*hydB*) were used to measure hydrogenase activity using a methyl viologen assay. The rate at which H_2_ was oxidized (in μmol/min) was obtained using the slope of absorbance at A_578 nm_, which was normalized to the amount of total protein in the cell lysate (in μg), and normalized against the activity of the WT strain to obtain percent hydrogenase activity. Three biological replicates were tested for each strain, and the mean and standard deviation are graphed. (B) Cell lysates of the WT, Δ*ureB*, and Δ*hydB* strains were used to measure urease activity using ammonia production in a phenol-hypochlorite assay. The specific urease activity was normalized to the amount of total protein in the cell lysate (in μg), and normalized against the specific activity of the WT strain to obtain relative urease activity. Three biological replicates were tested for each strain, and the mean and standard deviation are graphed.

In *Escherichia coli* K-12 [41, 42], *Salmonella enterica* serovar Typhimurium [43], and *Shigella flexneri* [44], hydrogenase mutant strains exhibit impaired acid resistance. However, to our knowledge the role of *H*. *pylori* hydrogenase in acid resistance has not yet been investigated. Thus, to determine the role of the hydrogenase pathway in *H*. *pylori* acid resistance, we generated a deletion mutation of *hydB* in the G27 strain background. To confirm that the Δ*hydB* strain was hydrogenase negative, hydrogenase activity was measured using a methyl viologen assay; the parental WT strain and a urease mutant strain (Δ*ureB*) were included as controls (Fig 2A). As expected [17], the Δ*hydB* strain was hydrogenase negative (Fig 2A). Unexpectedly, the Δ*ureB* strain showed increased hydrogenase activity as compared to that of WT. To confirm that deletion of hydrogenase had no effect on urease activity as previously described [45], urease activity was measured using a modified phenol-hypochlorite assay for NH_3_ production. The Δ*hydB* strain exhibited urease activity similar to the wild-type strain (Fig 2B).

**Fig 3:**
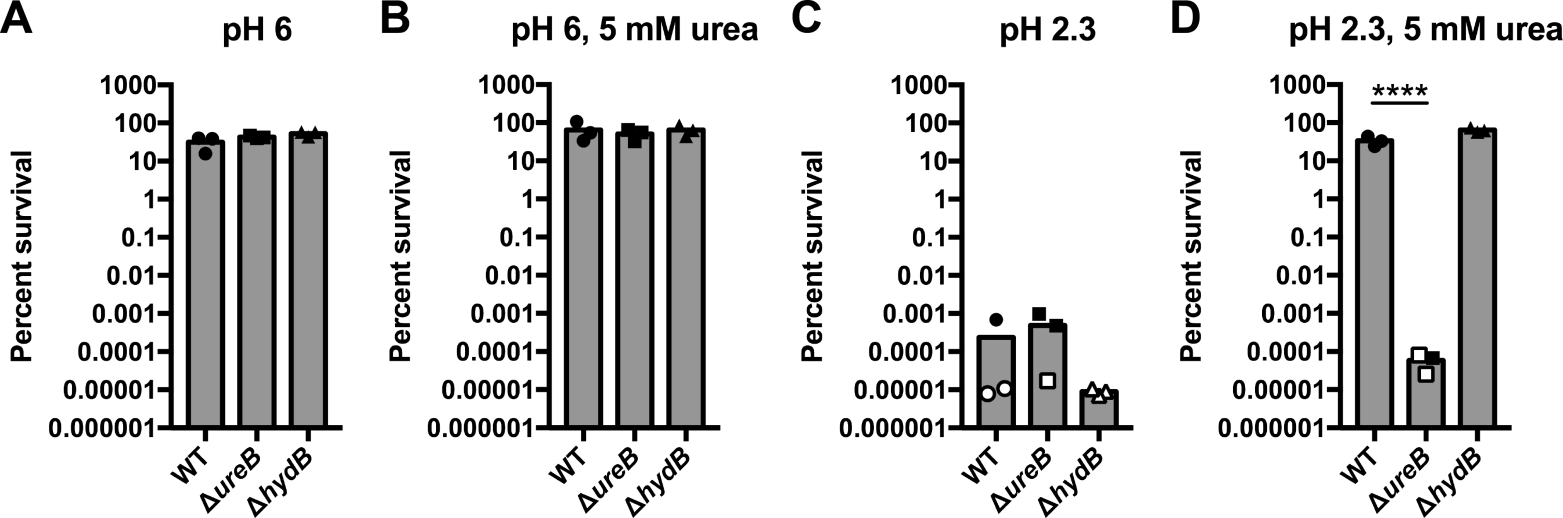
The Δ*hydB* strain of *H*. *pylori* G27 is not attenuated for acid survival. The wild-type (WT) strain, urease mutant strain (Δ*ureB*), and hydrogenase mutant strain (Δ*hydB*) were incubated for 1 hr in PBS adjusted to pH 6.0 (A and B) or to pH 2.3 (C and D), in the absence (A and C) or presence (B and D) of 5 mM urea. The number of colony-forming units (CFU) was measured at 0 min (T_0_) and at 60 min (T_60_), and percent survival was calculated as CFU at T_60_ divided by CFU at T_0_. Data from individual biological replicates are shown as points, with the bar plotted at the mean. Open symbols indicate that no bacteria were recovered and thus, the CFU are plotted as a function of the limit of detection (100 CFU/mL). Three biological replicates were performed. For panels A-C, a one-way ANOVA followed by Dunnett’s test for multiple comparisons was performed; the comparison was made only to WT. In panel D, the same statistical tests were performed on the log-transformed data. **** = p < 0.0001.

We next examined the contribution of hydrogenase to *H*. *pylori* acid resistance; bacteria were exposed to pH 6.0 or pH 2.3, in the presence or absence of 5 mM urea. All strains survived at pH 6.0, independent of the presence or absence of urea (Fig 3A and 3B). Conversely, at pH 2.3 without urea, all strains exhibited significantly impaired survival (limit of detection [LOD] = 100, open symbols, Fig 3C). At pH 2.3 in the presence of urea, the Δ*ureB* strain was extremely acid sensitive (Fig 3D); this was expected since the Δ*ureB* strain is unable to utilize urea to neutralize low pH. In contrast, survival of the WT and Δ*hydB* strains was comparable (Fig 3D). Thus, unlike in other pathogenic bacteria, hydrogenase appears to play no role in acid resistance in *H*. *pylori*.

**Fig 4:**
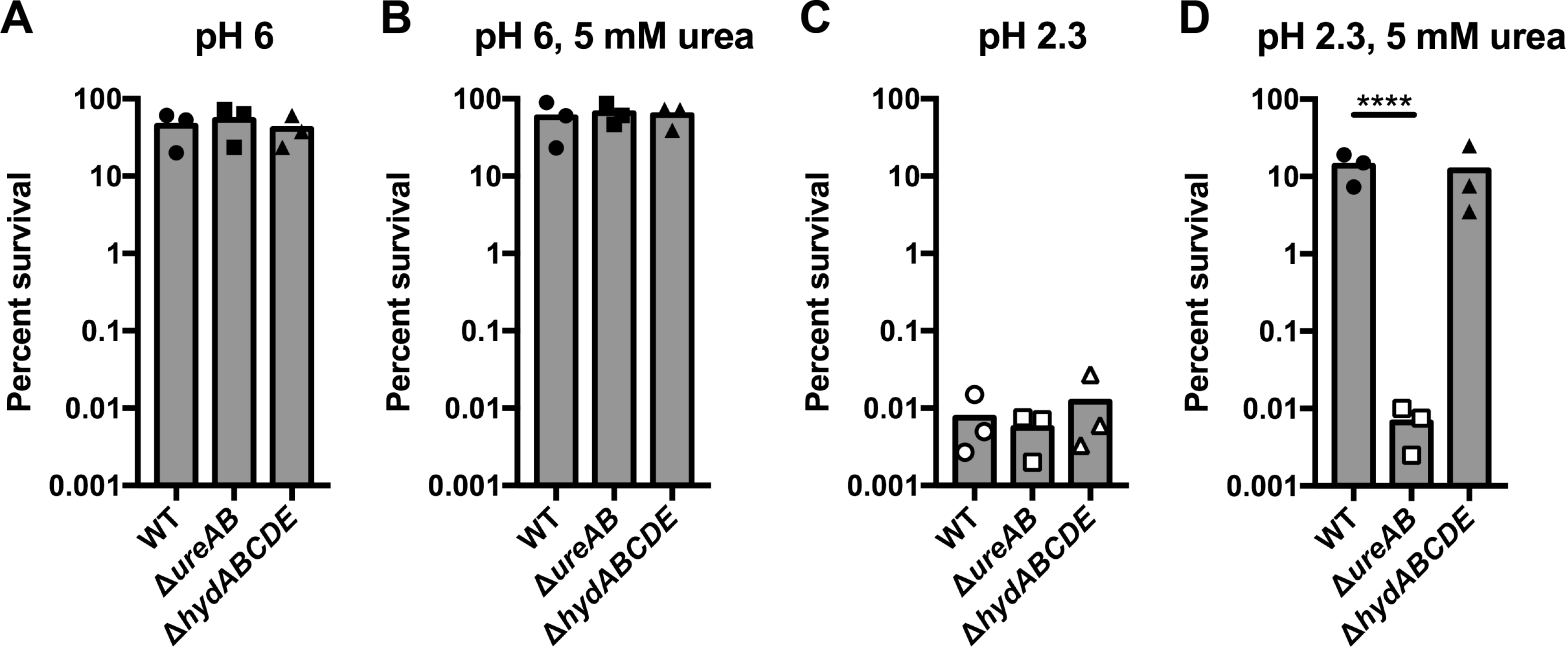
The Δ*hydABCDE* strain of *H*. *pylori* 26695 is not attenuated for acid survival. The wild-type (WT) strain, urease mutant strain (Δ*ureAB*), and hydrogenase mutant strain (Δ*hydABCDE*) were incubated for 1 hr in PBS adjusted to pH 6.0 (A and B) or to pH 2.3 (C and D), in the absence (A and C) or presence (B and D) of 5 mM urea. The number of colony-forming units (CFU) was measured at 0 min (T_0_) and at 60 min (T_60_), and percent survival was calculated as CFU at T_60_ divided by CFU at T_0_. Data from individual biological replicates are shown as points, with the bar plotted at the mean. Open symbols indicate that no bacteria were recovered and thus, the CFU are plotted as a function of the limit of detection (1000 CFU/mL). Three biological replicates were performed. For panels A-C, a one-way ANOVA followed by Dunnett’s test for multiple comparisons was performed; the comparison was made only to WT. In panel D, the same statistical tests were performed on the log-transformed data. **** = p < 0.0001.

Given the high degree of genetic diversity seen between strains of *H*. *pylori* [46, 47], we next sought to confirm these results in an additional *H*. *pylori* strain. To this end, we utilized a Δ*hydABCDE* mutant strain [32], and constructed a urease-negative control (Δ*ureAB*), in *H*. *pylori* 26695. As shown in Fig 4, the results obtained with the 26695 strain background were comparable to those obtained with G27. *En masse*, these data indicate that hydrogenase does not contribute to *in vitro* acid resistance of *H*. *pylori*.

### Metal coordination in HypA is necessary for hydrogenase activity

We previously constructed and characterized a panel of isogenic strains of *H*. *pylori* G27 that contained mutations at the zinc and nickel coordination sites of *hypA* [24, 26, 30]. Within the zinc-binding site, the four Cys residues (C74, C77, C91, and C94) were mutated to Ala and Asp, and the two His residues (H79 and H95) were mutated to Ala; mutation of any of these Cys residues results in a protein that at neutral pH adopts a conformation that features a Zn(Cys)_2_(His)_2_ site, similar to the conformation favored by WT-HypA in an acidic environment [24]. Likewise, mutation of either His residue within the zinc-binding site results in a Zn(Cys)_4_ structure, similar to that adopted by WT-HypA in a neutral environment [24]. Within the nickel-binding site, a Leu residue was inserted into position 2 (L2*), between the Met and His residues of the invariant MHE motif of the HypA-HybF protein family [48]; this insertion extends the distance to the N-terminal amine on the Met residue and changes the coordination and electronic structure of nickel at this site [26].

**Fig 5:**
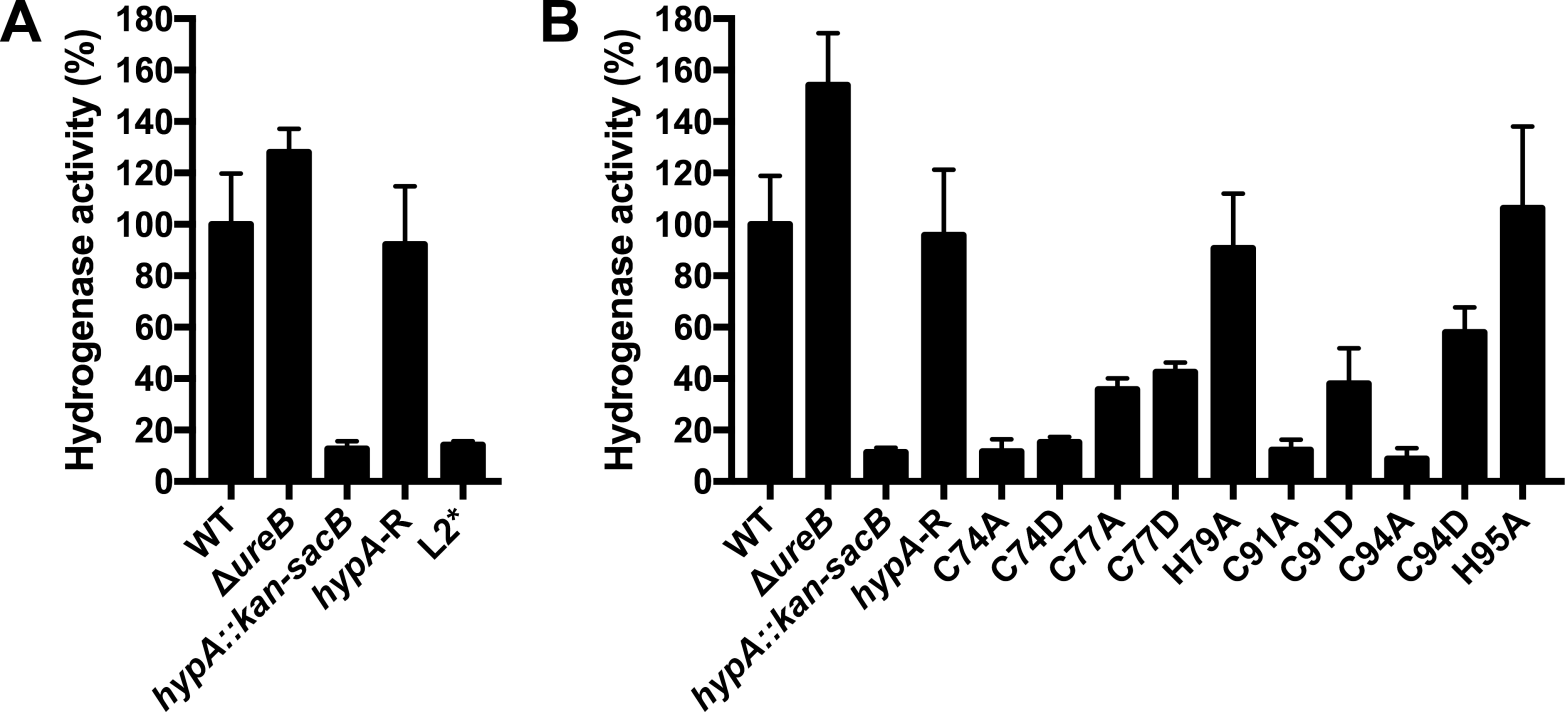
Mutation of the metal coordination sites of HypA results in decreased hydrogenase activity. Cell lysates from the indicated *hypA* mutant strains, in addition to wild-type (WT) strain, urease mutant strain (Δ*ureB*), *hypA* mutant strain (*hypA*::*kan-sacB*), and *hypA* restorant (*hypA*-R) were utilized to determine hydrogenase activity using a methyl viologen assay. The rate at which H_2_ was oxidized (in μmol/min) was obtained using the slope of absorbance at A_578 nm_, which was normalized to the amount of total protein in the cell lysate (in μg), and normalized against the activity of the WT strain to obtain percent hydrogenase activity. The hydrogenase activities of *hypA* mutant strains with mutations found within the nickel-binding site (A) and within the zinc-binding site (B) are shown. Two biological replicates were tested in A, and three biological replicates were tested in B. The mean is graphed, with range (A) or standard deviation (B).

This panel of HypA mutations was previously used to examine the contribution of the metal coordination sites to urease maturation and acid resistance [24, 26, 30]. However, a functional role for these changes to the HypA nickel- and zinc-binding sites has not been examined for hydrogenase maturation. Therefore, we used these isogenic strains to measure hydrogenase activity in comparison to a series of control strains; the control strains included the WT strain, Δ*ureB*, *hypA*::*kan-sacB*, and a *hypA* restorant (*hypA*-R) strain that was created to control for potential issues related to genetic manipulation of *H*. *pylori*. Analysis of the strains showed that the hydrogenase activity of the L2*-HypA mutant strain was reduced to approximately 14% of WT activity (Fig 5A). This was comparable to the hydrogenase activity observed with the *hypA*::*kan-sacB* mutant strain (12%). Similar to the results shown in Fig 2A, the hydrogenase activity of the Δ*ureB* strain was higher than in the parental WT strain (Fig 5A and 5B). Mutations within the zinc-binding site resulted in a variety of effects on hydrogenase activity (Fig 5B). While mutation of either His residue (H79 and H95) had little effect on hydrogenase activity (90 and 110%, respectively), the remaining mutations could be categorized into those with moderate decreases and severe decreases in hydrogenase activity. Moderate decreases were observed for C77A (36%), C77D (43%), C91D (40%), and C94D (60%). Severe decreases were observed for C74A (12%), C74D (15%), C91A (12%), and C94A (9%); these values were similar to those observed in the *hypA*::*kan-sacB* strain (12%). Thus, though the nickel and zinc coordination sites of HypA are critical for the hydrogenase maturation pathway, the absolute importance of each residue varies to some degree.

Though the effect of individual mutations within the metal-coordinating sites of HypA appears to be consistent between urease [26, 30] and hydrogenase activities (Fig 5), we wanted to compare these values more directly. Therefore we compiled data for the enzyme activities, acid survival, and nickel dissociation constants from the current study and previous publications [24, 26, 30] and present them in Table 2. Mutation of the His residues within the zinc-binding site had little effect on hydrogenase activity, urease activity, or acid survival, suggesting that HypA locked into the neutral pH-structure still contributes fully to urease and hydrogenase maturation. On the other end of the spectrum, the L2*-HypA mutation within the nickel-binding site had a dramatic effect on enzymatic activities and on acid survival, often to the same level as deletion of *hypA*. This suggests that either the decrease in nickel affinity or change in nickel coordination disrupts the ability of HypA to contribute to hydrogenase and urease maturation. Phenotypes associated with mutation of the Cys residues within the zinc-binding site were variable, but the mutations with the largest effect on acid survival (C91A and C94A) also had severe effects on urease and hydrogenase activity. Similarly, HypA mutants C77A, C77D, C91D, and C94D, which displayed moderate decreases in urease activity (2.9, 5, 5, and 5%, respectively), presented moderate decreases in hydrogenase activity (36, 43, 40, and 60%, respectively), and had little/no effect on acid survival (90, 90, 90, and 110%, respectively). Thus, any HypA mutation locked into the acidic pH-structure resulted in some degree of defect in downstream enzymatic maturation. Of note, for mutations within the zinc-binding site, nickel affinity only roughly correlated to hydrogenase or urease activity. The two His mutants retained nickel affinity similar to WT HypA, and had little effect on hydrogenase or urease activities. Where the nickel affinity of the Cys mutants is known, all have a *K*_D_ 10-fold higher than WT HypA, and some effect on hydrogenase and urease activity; however, within the Cys mutants, we could not directly correlate nickel affinity to the downstream enzymatic activities. While the reason for this is not immediately evident, we do note that the *K*_D_ values are measured using purified proteins and may not accurately reflect what is seen within the *H*. *pylori* cell. Further, we note that it is possible that the mutation of individual HypA residues may affect the level of HypA that accumulates within the cell, which could impact downstream hydrogenase and urease activities. Future studies will be required to determine if these mutations affect the stability and steady-state concentration of HypA within *H*. *pylori*. Overall, these data indicate that HypA-dependent maturation of both urease and hydrogenase requires identical residues and similar coordination at the zinc- and nickel-binding sites.

## Discussion

Though a role for hydrogenase in acid resistance has been shown in several other pathogenic bacteria, the [NiFe] uptake-type hydrogenase of *H*. *pylori* does not appear to contribute to acid resistance (Figs 1B, 3 and 4). Though the reason for this variation across pathogens is not completely clear, several interesting differences across the various species are apparent. For example, *Salmonella enterica* serovar Typhimurium carries three hydrogen uptake-type hydrogenases (*hya*, *hyb* and *hyd*), in addition to two hydrogen-evolving hydrogenases (*hyc* and *hyf*). Deletion of *hya*, but not *hyb* or *hyd*, results in loss of acid resistance and impaired survival inside macrophages [43]. Though the exact mechanism by which Hya contributes to the acid stress response of *S*. Typhimurium is unknown, a mechanism involving energy conservation by Hya via recycling of H_2_ produced by Hyc has been proposed [43, 49]. Interestingly, deletion of the three uptake hydrogenases (*hya*, *hyb*, and *hyd*) results in a colonization-deficient strain of *S*. Typhimurium; furthermore, each double mutant strain was less virulent than the parent strain [50]. Similarly, in *Shigella flexneri*, deletion of the hydrogen-uptake-type hydrogenase *hya* results in an acid sensitive mutant strain [44], while in *Escherichia coli*, deletion of the hydrogen-evolving hydrogenase encoded by *hyc* results in acid sensitivity in anaerobic growth conditions [42]. It is worth noting that each of these other pathogens that rely on a hydrogenase for acid resistance have limited mechanisms for dealing with acid stress as compared to urease activity in *H*. *pylori*. Indeed, acid neutralization is accomplished quickly by *H*. *pylori* urease; when incubated in unbuffered HCl as low as pH 3, *H*. *pylori* increases the pH to neutral within 1 min [11]. Despite living in the human stomach, *H*. *pylori* is not an acidophile, and appears to synthesize a large amount of urease (up to 10% of the nascent protein content) for protection against a sudden decrease in pH [11]. With such a robust mechanism for acid neutralization, and so much energy committed to urease synthesis and nickel sequestration, perhaps *H*. *pylori* simply does not need to dedicate a second nickel-containing enzyme (hydrogenase) to cope with acid stress.

We observed a loss of acid resistance in strains of *H*. *pylori* lacking urease, but not in strains lacking hydrogenase (Figs 3 and 4). Previous studies have shown that hydrogenase mutant strains of *H*. *pylori* are deficient for colonization in mouse and Mongolian gerbil models of infection [7, 8]. The degree of deficiency varies depending on the animal model and/or *H*. *pylori* strain background [7, 8]. Given the lack of a pH-sensitive phenotype, the role of hydrogenase during *H*. *pylori* animal infection may solely be the previously proposed utilization of H_2_ as an energy source [7]; this role is intricately linked to powering of DNA uptake through the ComB type IV secretion system (T4SS) and CagA toxin translocation through the Cag T4SS [8]. Decreased CagA translocation is supported by the fact that gerbils infected with a Δ*hyd* mutant strain of *H*. *pylori* exhibited lower levels of inflammation [8]. Interestingly, hydrogenase mutant strains of *Helicobacter hepaticus* do not induce inflammation or necrosis in a model of liver infection in mice [51]. Thus, perhaps the energy produced by hydrogenase plays a more global role in the synthesis of downstream virulence factors in a number of different pathogenic bacteria.

While mutation of hydrogenase or urease resulted in the expected loss of each respective enzymatic activity (Fig 2, [30]), we unexpectedly found that deletion of *ureB* consistently resulted in an increase in hydrogenase activity above that seen in the wild-type parental strain (Figs 2A, 5A and 5B). Interestingly, the reverse did not hold true; deletion of hydrogenase resulted in only a slight increase (~12%) in urease activity in the G27 background (Fig 2B), as was previously observed in the 26695 background [45]. Both the urease and the hydrogenase maturation pathways require intracellular nickel and the accessory proteins HypA and HypB. However, urease is the major nickel sink [13], suggesting that the equilibrium between the two pathways favors urease. We hypothesize that deletion of urease increases the nickel available to the hydrogenase maturation pathway, resulting in an increase in hydrogenase activity. While deletion of hydrogenase also increases the nickel available to the urease maturation pathway, it is to a lesser extent; therefore, this results in only a slight increase in urease activity.

The N-terminal nickel-binding site of HypA requires both the N-terminal amine [26] and His2 [23]. Indeed, the localization of the nickel-binding site to the N terminus was originally determined based on the loss of nickel binding in a H2A-HypA mutant strain [23]. It is worth noting that the H2A-HypA and L2*-HypA mutations share several characteristics, each of which suggest that loss of the imidazole or N-terminal amine as a nickel ligand results in similarly deficient HypA proteins. For example, though neither mutation causes overt structural changes, both are deficient for nickel binding [23, 26]. Both H2A-HypA and L2*-HypA retain protein-protein interactions with the partners that have been tested: cross-linking reactions showed that H2A-HypA interacts with HypB [23] and isothermal calorimetry studies showed that L2*-HypA interacts with UreE [26]. Urease activity of each mutant strain is greatly reduced: ~2% for H2A-HypA [23] and 0.1% for L2*-HypA [26]. Furthermore, hydrogenase activity was also impaired for each mutant strain: <2.5% for H2A-HypA [23] and 14% for L2*-HypA (Fig 5A). Though not tested with H2A-HypA, studies indicate that the zinc-binding site is unaffected in L2*-HypA, and that the coordination around the nickel ion changes from 6-coordinate to 5-coordinate geometry, which causes the Ni site to adopt a low-spin electronic configuration [26]. Taken together, the available data suggest that mutation of the HypA nickel-binding site does not affect the protein-protein interactions involved in downstream urease and hydrogenase maturation, but instead results in a decrease in urease and hydrogenase activities due to a decrease in HypA nickel affinity and subsequent inability of HypA to pass nickel to each maturation pathway.

Within the zinc-binding site and surrounding residues of HypA, it is worth noting that the various constructed mutations show dramatically different phenotypes. The His→Ala mutations resulted in urease activity, hydrogenase activity, and acid survival similar to WT-HypA (Fig 5B, Table 2, [30]). Prior studies indicate that the His→Ala mutations result in a structure similar to that which WT-HypA adopts at neutral pH [24]. Furthermore, the zinc site remained Zn(Cys)_4_ at both neutral and acidic pH. Finally, the His mutants show the same affinity for nickel as WT-HypA; this includes a weaker affinity for nickel at low pH as compared to neutral pH [24]. The relatively dispensable role for the His residues stands in stark contrast to the role played by the Cys residues in the CxxC motifs. In regards to zinc coordination, nickel affinity, and the amount of nickel bound, mutant HypA containing either Cys→Ala or Cys→Asp mutations adopts the conformation similar to that of WT-HypA in an acidic environment; mutation of a single Cys residue caused the zinc site to switch to a Zn(Cys)_2_(His)_2_ conformation, which was maintained regardless of pH [24]. These Cys mutations also result in a weaker affinity for nickel at neutral pH [24]. In terms of urease activity, acid survival, and hydrogenase activity (Fig 5, Table 2, [30]), three broad categories of CxxC mutants can be delineated based on the associated phenotypes. First, three of the four Cys→Asp mutants, C77D, C91D, and C94D, as well as the C77A mutant, caused a moderate defect to urease and hydrogenase activity, yet displayed acid resistance equal to WT-HypA. Second, the C74A and C74D mutants were severely defective for urease and hydrogenase activity, but had little effect or a moderate effect, respectively, on acid resistance. Last, the C91A and C94A mutants displayed severe defects in urease and hydrogenase activity, and in acid resistance. No mutants within the panel of HypA mutations displayed a defect in acid resistance without also having a concomitant severe defect in urease and hydrogenase activity. However, a severe defect in urease activity was not always predictive of survival in low pH; for example, mutants C74A, C74D, and C94A showed similar hydrogenase and urease activities, but no defect, a moderate defect, or a severe defect in acid resistance, respectively. This lack of predictive ability around a certain threshold of urease activity is likely due to the limited sensitivity of the assay in lysates containing little urease activity; the activity of even the Δ*ureB* strain was 0.7%. Thus, a more sensitive assay is needed to conclusively predict acid sensitivity. The affinity of the HypA Cys mutant proteins for nickel was not predictive of hydrogenase activity, urease activity, or acid resistance (Table 2, [24]). Instead, it is likely that within the intact *H*. *pylori* cell, it is the change in dynamics and/or zinc coordination in the HypA Cys mutant proteins [24] that is responsible for the decrease in hydrogenase and urease activities. Overall, hydrogenase and urease activities were similarly impaired by each HypA mutation. Thus, these findings support the idea that HypA-dependent delivery of nickel to the urease and hydrogenase pathways involves similar mechanisms and structural features of HypA.

In summary, the data presented herein demonstrate that the hydrogenase of *H*. *pylori* does not contribute to acid resistance. Furthermore, based on the data presented here and previously [26, 30], mutation of individual residues within the zinc- and nickel-binding sites of HypA affect urease and hydrogenase activity comparably. Thus, we conclude that the metal coordination sites of HypA play similar roles in urease and hydrogenase maturation.
